# Glycine-Group-Functionalized Polymeric Materials Impregnated with Zn(II) Used in the Photocatalytic Degradation of Congo Red Dye

**DOI:** 10.3390/polym17050641

**Published:** 2025-02-27

**Authors:** Laura Cocheci, Aurelia Visa, Bianca Maranescu, Lavinia Lupa, Aniela Pop, Ecaterina Stela Dragan, Adriana Popa

**Affiliations:** 1 Faculty of Chemical Engineering, Biotechnology and Environmental Protection, Politehnica University Timisoara, 6 Vasile Parvan Blvd., 300223 Timisoara, Romania; lavinia.lupa@upt.ro (L.L.); aniela.pop@upt.ro (A.P.); 2 “Coriolan Drăgulescu” Institute of Chemistry, 24 Mihai Viteazu Blvd., 300223 Timisoara, Romania; 3 Department of Chemistry, Faculty of Chemistry, Biology, Geography, West University Timisoara, 16 Pestalozzi Street, 300115 Timisoara, Romania; biancamaranescu@yahoo.com; 4 Petru Poni Institute of Macromolecular Chemistry, 41A Aleea Grigore Ghica Voda, 700487 Iasi, Romania; stela_dragan@yahoo.com

**Keywords:** glycine, styrene-divinylbenzene copolymer, heterogeneous photocatalyst, Congo red, photocatalysis

## Abstract

Reducing the ecological impact of dyes through wastewater discharge into the environment is a challenge that must be addressed in textile wastewater pollution prevention. Congo red (CR) dye is widely used in experimental studies for textile wastewater treatment due to its high organic loads used in its preparation. The degradation of organic dyes of the CR type was investigated using the photocatalytic activity of functionalized polymers. We have employed photodegradation procedures for both polymer-supported glycine groups (Code: AP2) and polymer-supported glycine-Zn(II) (Code: AP2-Zn(II)). A photocatalysis efficiency of 89.2% was achieved for glycine pendant groups grafted on styrene-6.7% divinylbenzene copolymer (AP2) and 95.4% for the AP2-Zn(II) sample by using an initial concentration of CR of 15 mg/L, a catalyst concentration of 1 g/L, and 240 min of photocatalysis. The findings provided here have shown that the two materials (AP2 and AP2-Zn(II)) may be effectively employed in the heterogeneous photocatalysis method to remove CR from water. From the perspective of the degradation mechanism of CR, the two photocatalysts act similarly.

## 1. Introduction

Organic contaminants such as textile dyes can pollute the environment, which poses a serious risk. Because of the dyes used and the chemicals added during preparation, wastewater from the textile sector has a significant organic load. The ability of the organic compounds to degrade dyes through photocatalysis was investigated. Processors are being compelled to reclaim and recycle process water and chemicals due to regulations limiting wastewater discharge into the environment and controlling water contamination, which may have an ecotoxicological impact [1,2]. A potential class of adsorbents is represented by cross-linked polymers because they can form a large variety of porous shapes inside a particular chemical system. Enhancing the surface chemistry of polymer supports to achieve superior adsorption capabilities against certain contaminants is a commonly employed method that involves chemically altering polymer matrices with pendant functional groups [3]. Considerable attention has been paid to polymeric matrices with pendant functional groups as foundational matrices for heterogeneous photocatalyst design [4]. A serious ecological issue has arisen as a result of the industrial sector’s use of a wider range of artificial dyes that are harmful to the environment in recent years [5,6]. Azo dyes are used extensively in the textile industry due to their strong washing resilience and ease of dyeing processes. These reactive dyes, which are identified by the azo bond (−N=N−) chromophores, are stable and non-biodegradable, making them challenging to remove with conventional treatment methods [7,8]. The dye Congo red (CR), one of the azo dyes, is frequently utilized as a model dye in studies on wastewater treatment related to textile dyeing [9,10]. Despite its widespread use, CR is inherently toxic to living organisms [9,10,11]. For sustainable growth, CR from wastewater must be eliminated using eco-friendly methods. Adsorbents with a positive charge on their surface are advantageous for CR adsorption because CR molecules are negatively charged in aqueous solutions. Both natural and synthetic polymers are employed as CR adsorption agents [11,12,13].

The use of heterogeneous photocatalysis to degrade textile dyes from wastewater has gained increasing attention in the past several years. Heterogeneous photocatalysis is a significant method for the filtration and repurposing of aqueous effluents. It involves exposing various dyes to UV light in the presence of a solid catalyst in wastewater [14,15]. In the present study, two novel glycine-type compounds with pendant groups grafted on S-DVB copolymer were used for the decontamination of CR-polluted water. They were characterized by FTIR spectroscopy, scanning electron microscopy, EDX spectroscopy, thermal analysis, and textural characterization.

We used photocatalysis in our photodegradation process for both polymer-supported glycine groups (Code: AP2) and polymer-supported glycine-Zn(II) (Code: AP2-Zn(II)). The photocatalytic activity of functionalized polymers was explored for the degradation of CR-type organic dyes. By using an initial concentration of 15 mg/L of CR and a catalyst concentration of 1 g/L, and with 240 min of irradiation, a photocatalysis efficiency of 89.2% in the case of glycine pendant groups grafted on styrene-6.7% divinylbenzene copolymer (AP2) and of 95.4% in the case of glycine-Zn(II) pendant groups grafted on styrene-6.7% divinylbenzene copolymer (AP2-Zn(II)), respectively, was achieved.

## 2. Materials and Methods

### 2.1. Materials and Equipments

Chloromethylated styrene-6.7% divinylbenzene copolymers (S-6.7%DVBCH_2_Cl, 4.01 mmol Cl/g copolymer)—material received from the Purolite, Victoria, Romania, glycine (aminoethanoic acid, NH_2_CH_2_COOH), ethanol (Chimreactiv, p.a.), and zinc nitrate tetrahydrate (Zn(NO_3_)_2_ × 4H_2_O, Sigma-Aldrich, Darmstadt, Germany) were used as starting materials for the preparation of the functionalized copolymers. The chemical structure of the pristine S-6.7%DVBCH_2_Cl and of the copolymer modified with glycine (AP2) was investigated first by FTIR spectroscopy (KBr pellets) on a JASCO FTIR spectrophotometer (JASCO, Tokyo, Japan) in the range 4000–400 cm^−1^ and the maximum resolution of FT-IR spectra of 0.4 cm^−1^.

The thermal properties of AP2 and AP2-Zn(II) products were characterized by thermogravimetric analysis on a TGA/SDTA 851-LF1100—Mettler-Toledo (Greifensee, Switzerland) equipment at temperature range from 25 to 900 °C and at a heating rate of 10 °C/min under nitrogen atmosphere. The specific surface area (S_sp_) and pore size distribution were estimated from N_2_ adsorption–desorption experiments conducted at 77 K using an Autosorb-1-MP surface area analyzer (Quantachrome Company, Boynton beach, FL, USA). Before measurements, the sample was outgassed at room temperature for two hours. The isotherms of nitrogen adsorption/desorption were measured at the relative pressure p/p_o_ in the domain 0.01–1.0. The S_sp_ was evaluated from the linear portion of the adsorption isotherms (0.05–0.35) by Brunauer–Emmett–Teller (BET) method. The Barrett–Joyner–Halenda (BJH) theory was used to determine the pore size distribution from the nitrogen adsorbed volume at saturation, respectively, at the relative pressure p/p_o_ nearest to unit (0.95).

The external surface and internal morphology of the copolymer, before and after the reaction with glycine, were observed by using an Environmental Scanning Electron Microscope (ESEM)-type (FEI Company, Hillsboro, OR, USA) Quanta 200, operating at 20 kV with secondary electrons, in low vacuum mode. The elemental composition of the copolymers microspheres was evaluated with an Energy-Dispersive X-ray (EDX) analyzer (Octane Elect Super SDD detector, Ametek, Berwyn, PA, USA) equipped on a Verios G4 UC (Thermo Scientific, Brno, Czech Republic) SEM device. UV–Vis diffuse reflectance spectra were recorded by using a Varian Carry 100 spectrophotometer equipped with an integrating diffuse reflectance sphere. By applying the Kubelka–Munk equation to the response, the band-gap energies were deduced. Photocatalysis was carried out using a PhotoLAB B400-700 Basic Batch-L photoreactor supplied by Peschl-Ultraviolet GmbH (Mainz, Germany) and equipped with a submerged lamp (150 W Hg medium pressure, type TQ150) that has emission both in UV and Vis domain. During the operation of the photoreactor, the temperature of the system is kept constant, at about 20 °C, by the cooling water from the outer jacket of the reaction vessel.

### 2.2. Obtaining of Copolymer Functionalized with Glycine (AP2)

**Scheme 1:**
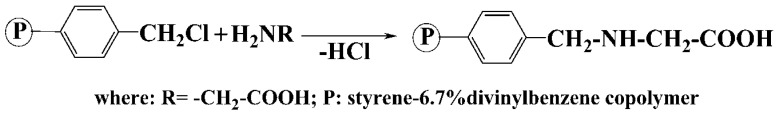
Obtaining of glycine functionalized onto copolymer (AP2).

In a flask equipped with a thermometer, stirrer, and refrigerant for reflux, the copolymer S-6.7%DVBCH_2_Cl (10 g) and glycine were introduced at a molar ratio of 1:1 compared to the pendant group (-CH_2_Cl). Glycine was previously dissolved in 100 mL/30 mL EtOH/H_2_O solution. The synthesis took place for 30 h at a temperature of 78 °C. The final product was filtered, washed with hot distilled water and ethanol, and dried at 50 °C for 24 h. Scheme 1 shows how the AP2 product was obtained.

### 2.3. Obtaining AP2-Zn(II)) Sample

Four grams of copolymer S-6.7%DVB functionalized with glycine (AP2) was stirred for swelling in 50 mL of ethanol. Then, 1.07 g Zn(NO_3_)_2_ × 4H_2_O previously dissolved in 25 mL of ethanol was added. The synthesis was carried out in an ultrasonic bath at 45 °C. After 20 h, the flask was removed from the ultrasound bath, and the product was filtered. Sample was denominated AP2-Zn(II).

### 2.4. Photocatalysis

Photocatalytic experiments were performed by contacting a specific mass of photocatalyst with 700 cm^3^ of CR solution with a certain initial concentration so that the solid–liquid ratios became 0.5, 1, and 2 g/L. The system was magnetically stirred in the dark for 30 min to achieve equilibrium; then, the lamp was turned on, and, at different time intervals, a small aliquot of 5 cm^3^ was extracted and spectrophotometrically analyzed. The working pH was the natural pH of the CR solutions. The dye concentration was determined based on the molecular spectrometry Lambert–Beer law by reading the absorbance of the solution at λ_max_ = 498 nm. The experiments were conducted in triplicate, and the results presented are the averages of the obtained values. The efficiency of CR removal (*R*) was calculated using the following formula:

(1) $$R\left(\%\right)=\frac{c_{0}-c}{c_{0}}\times 100$$

where *c*_0_ is the initial concentration of CR (mg/L), and *c* is the concentration of CR at time *t* (mg/L).

### 2.5. Design of Experiments and Optimization by Response Surface Methodology

The optimization of experimental data collection in order to maximize the efficiency of the photocatalytic process was achieved using the Response Surface Methodology (RSM). The experimental design was built by applying the Box–Benhken design (BBD) model and developed using Stat-Ease^®^ 360 (version 23.1.8) software. In the BBD, the experimental points are located on a hypersphere, equidistant from the central point. Depending on the levels of the parameters chosen to be optimized, the number of experiments required is less than in the case of other models [16], and the maximum information on optimal conditions can be obtained with the minimum number of experiments. Choosing to optimize the efficiency of the photocatalysis process using 3 parameters and 3 levels, the answer of the modeling by the Box–Benhken design method is obtained in the form of a polynomial (quadratic) equation as follows [16]:

R (%) = b_0_ + b_A_A + b_B_B + b_c_C + b_AB_AB + b_AC_AC + b_BC_BC + b_A2_A^2^ + b_B2_B^2^ + b_C2_C^2^ (2)

where R (%) is the dependent factor or answer (efficiency), A, B, and C represent the independent variables in coded levels, b_0_ is intercept, and b_A_, b_B_, b_C_, b_AB_, b_AC_, b_BC_, b_A2_, b_B2_, and b_C2_ are the coefficients of the quadratic model.

Among the parameters that influence photocatalysis (the initial concentration of the dye, the dose of the catalyst, the pH of the reaction medium, and time), the following were chosen to be optimized: CR initial concentration, solid–liquid ratio, and time. After performing the experiments according to the matrix presented in Table 1, the results were modeled, and the significance of the model was evaluated using ANOVA. Table 1 presents the experimental levels of independent parameters utilized for RSM optimization.

## 3. Results and Discussion

### 3.1. Morphological and Chemical Characterization of AP2 Copolymer

**Figure 1:**
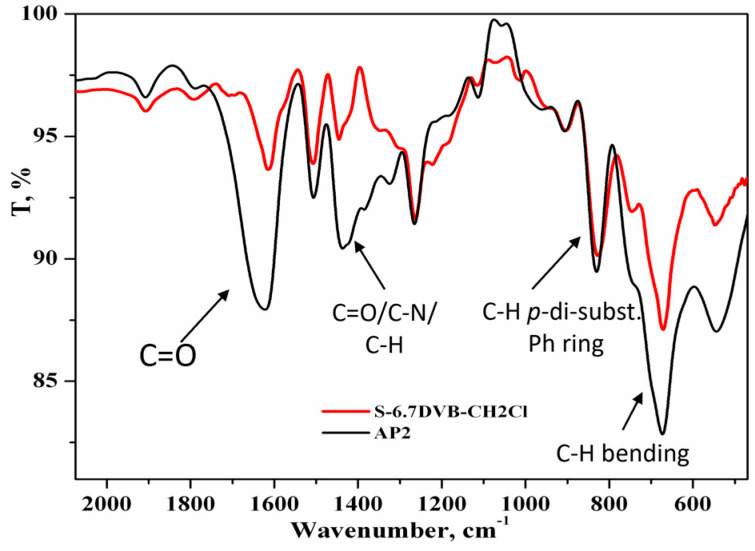
FTIR spectra.

**Figure 2:**
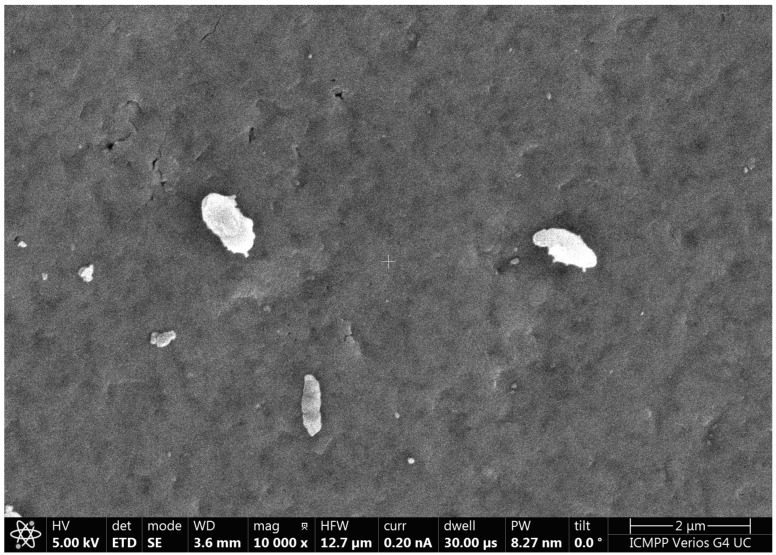
SEM image of AP2; mag. 10,000× and scaling bar 2 µm.

The FTIR spectra of the glycine-functionalized copolymer (AP2) and the pristine copolymer S-6.7DVB-CH_2_Cl are displayed in Figure 1. A slight increase for the adsorption band from 1621 cm^−1^ has been assigned to the stretching vibrations of the C=O [17], and it is shown in the spectrum for AP2. The higher intense broadband at 1421–1324 cm^−1^, which corresponds to CO/C-N/C-H, stretching/bending vibrations, suggests the presence of amino acids [18,19] and indicates that the copolymer has been functionalized. Figure 2 presents the SEM image of the copolymer AP2. As can be seen, the AP2 sample displayed a relatively smooth surface with some defects.

### 3.2. Textural Characteristics of the AP2 Copolymer

**Figure 3:**
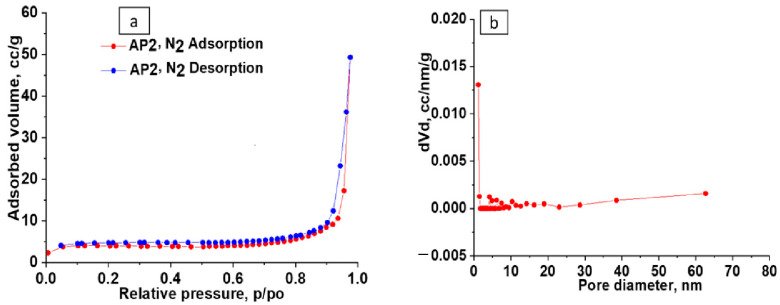
BET isotherm (**a**) and BJH distribution of pore diameter (**b**) of the AP2 copolymer.

The plots of BET isotherms and BJH distribution of pore diameters for the AP2 copolymer are presented in Figure 3.

The BET isotherm corresponds to type IV, indicating the uniformity and regularity of the textural properties of the AP2 copolymer. This type of isotherms is usually associated with capillary condensation in mesoporous structures [20,21,22]. The BET surface area of the AP2 copolymer, calculated by applying the BET equation to the linear part of the adsorption isotherm (0.05 < p/p_o_ < 0.305), is 15.856 m^2^/g. According to IUPAC classification, the pores in porous materials are classified into micropores (width less than 2 nm), mesopores (width between 2 and 50 nm), and macropores (width greater than 50 nm) [20]. The distribution of pore diameters in Figure 3b would indicate that the majority of pores are located in the range of 3–50 nm, and this shows a mesoporous structure for this copolymer.

### 3.3. Characterization of AP2 Copolymer Impregnated with Zn(II) (Code: AP2-Zn(II))

**Figure 4:**
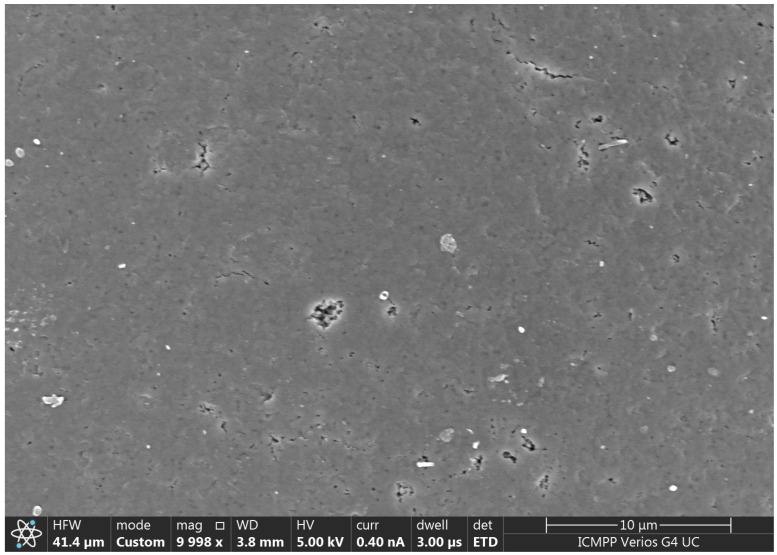
SEM images for AP2-Zn(II); mag. 9998× and scaling bar 10 µm.

As can be seen in Figure 4, the external surface of the microspheres was not influenced by the reaction of AP2 with glycine and looked clean with minor defects.

**Figure 5:**
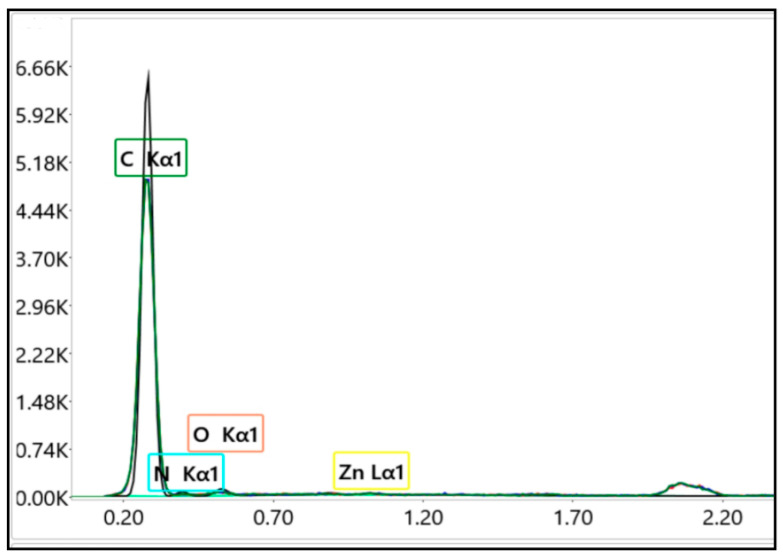
EDX spectrum of AP2-Zn(II).

**Figure 6:**
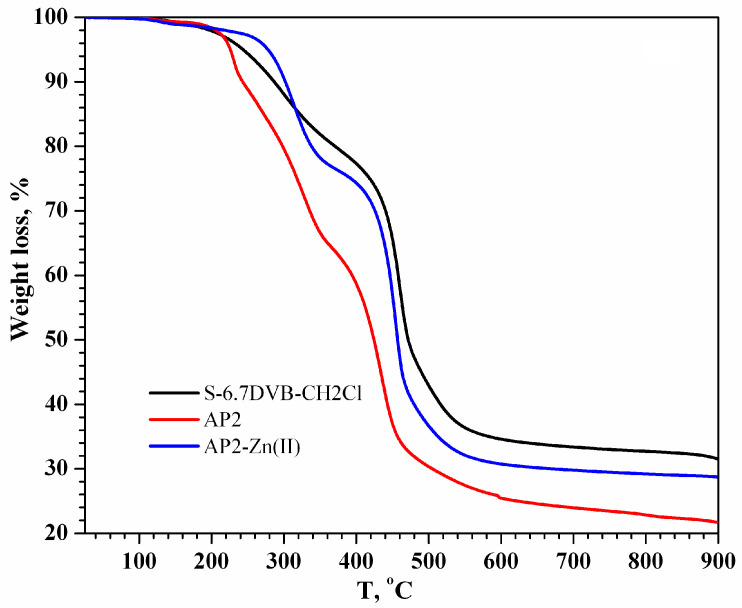
TG analysis for S-6.7DVB-CH2Cl, AP2, and AP2-Zn(II) samples recorded in nitrogen atmosphere.

The EDX spectrum presented in Figure 5 supports both the reaction of the styrene-6.7% DVB with glycine and the loading of AP2 with Zn(II). Thus, the average values (wt%) of the elements identified in AP2-Zn(II) are C = 91.79%, N = 4.72%, O = 2.85%, and Zn = 0.64%. The thermal behavior of samples of AP2, AP2-Zn(II), and pristine copolymer (S-6.7DVB-CH2Cl) can be seen in Figure 6. In Figure 6, the TG curves of the AP2 and AP2-Zn(II) samples were compared. Three temperature stages are represented in the TG curve for AP2. Weight loss in two temperature stages is displayed in the TG curve for AP2-Zn(II) (see Figure 6). The analysis of the samples showed the residues and weight losses at the final temperature of 900 °C: for the pristine copolymer, the residue was 31.58%, and the weight loss was 68.42%; for the AP2 copolymer, the residue was 21.72%, and the weight loss was 78.28%; and for the AP2-Zn(II) copolymer, the residue was 28.74%, and the weight loss was 71.26%. As such, the stability of the AP2-Zn(II) sample is greater.

The value 7.02% is the final residue, which corresponds to ZnO from the difference between the residues of AP2-Zn(II) and AP2 (28.74–21.72%) [23].

From the final residue value of ZnO (7.02%) was determined the yield of zinc impregnation on AP2 support as 84.3%.

### 3.4. UV–Vis Diffuse Reflectance Spectra and Band-Gap Energies

**Figure 7:**
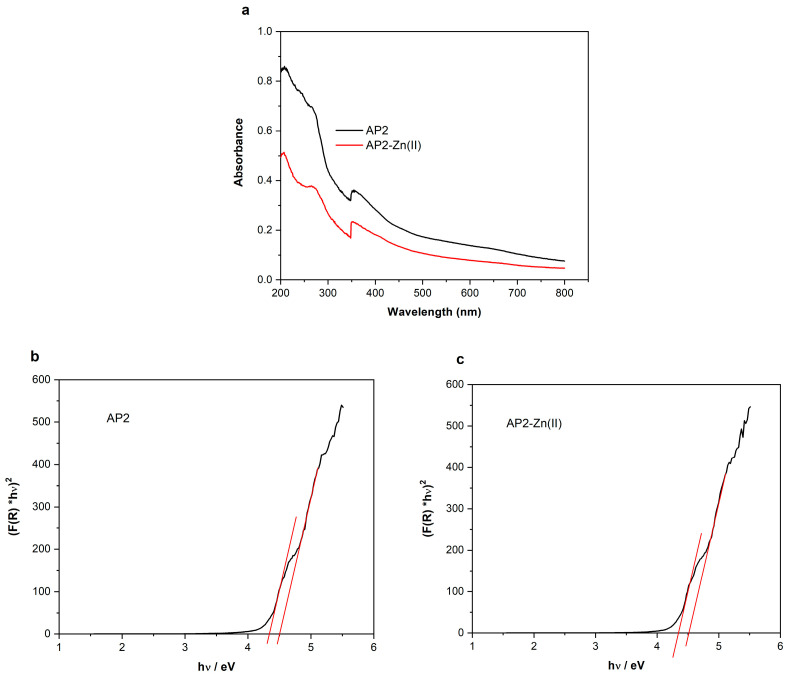
(**a**) UV–Vis diffuse reflectance spectra of the materials; (**b**) Kubelka–Munk plots for determination the band-gap energies of AP2 and (**c**) AP2-Zn(II).

The UV–Vis diffuse reflectance spectra are presented in Figure 7a. The absorption bands of the samples are similar, and both samples exhibit a wide absorption band in the UV range, indicating that the materials are photoabsorbent. The presence of three shoulders is observed, originating from the intra-aromatic π–π* transitions in the copolymer. The first two shoulders have the same maxima for both samples, at 210 nm and 275 nm. The AP2 sample displays the last absorption maximum at 370 nm, while the AP2-Zn(II) sample has the last absorption maximum at 350 nm. This hypsochromic effect present in the AP2-Zn(II) sample may be due to the presence of zinc.

The band-gap energies resulting from Kubelka–Munk transformations are 4.338 eV and 4.506 eV for AP2 and 4.354 eV and 4.523 eV for AP2-Zn(II), respectively. The values of the band-gap energies are close to each other, and it is expected that the behaviors of the two materials will recommend them as photocatalysts. Such band-gap energy values have been reported for polymers [24,25].

### 3.5. Optimization of Photocatalytic Tests

The matrix of the experimental design and the answer given by the BBD model, based on the experiments regarding the removal of CR by photocatalysis processes, are presented in Table S1.

Based on the experimental model designed by BBD, second-order polynomial equations were obtained in terms of the current factors. The equations show the dependence of the efficiencies of RC removal on the chosen independent parameters, as follows:

For the utilization of AP2 as photocatalysts,

R1 (%) = 77.10 − 10.54A + 16.89B + 5.75C − 4.03AB − 0.50AC + 3.70BC − 2.29A^2^ − 13.74B^2^ − 7.21C^2^ (3)

For AP2-Zn(II) utilized as photocatalysts,

R2 (%) = 81.90 − 9.04A + 16.73B + 4.69C − 2.38AB + 0.30AC + 3.53BC + 4.42A^2^ − 21.45B^2^ − 8.42C^2^ (4)

For Equation (3), the terms B, C, and BC have a positive effect on the process efficiency, while the other terms (A, AB, AC, A^2^, B^2^, and C^2^) have a negative effect.

For Equation (4), the terms B, C, AC, BC, and A^2^ have a synergistic effect and the terms A, AB, B^2^, and C^2^ have an antagonistic effect.

The adequacy of the quadratic design was verified by the analysis of variance (ANOVA). The ANOVA for the polynomial model results is presented in Table S2. For the two models, the F-values are 22.71 (AP2 sample) and 10.25 (AP2-Zn sample) with a *p*-value of 0.0002 for both models, which means that the two models are significant, with a 0.02% chance that the F-values can come from noise.

*p*-values less than 0.05 indicate that those terms are significant for the value of the regression coefficient, while terms with *p*-values greater than 0.01 indicate that they are not significant. For both samples, AP2 and AP2-Zn(II), utilized as a photocatalyst, the significant terms are A (the initial concentration of dye), B (solid–liquid ratio), C (time), and B^2^.

The R^2^ regression coefficients are 0.9669 and 0.9295, the adjusted R^2^ coefficients are 0.9243 and 0.8388, and the adequate precision are 15.0950 and 9.9477 for the AP2 and AP2-Zn(II) samples, respectively. Adequate precision is the signal-tonoise ratio, and aratio greater than 4 indicates an adequate signal. In conclusion, the use of the BBD-model approximates with sufficient accuracy the experimental data obtained from the research on the use of the two materials as photocatalysts for the removal of CR dye from water.

**Figure 8:**
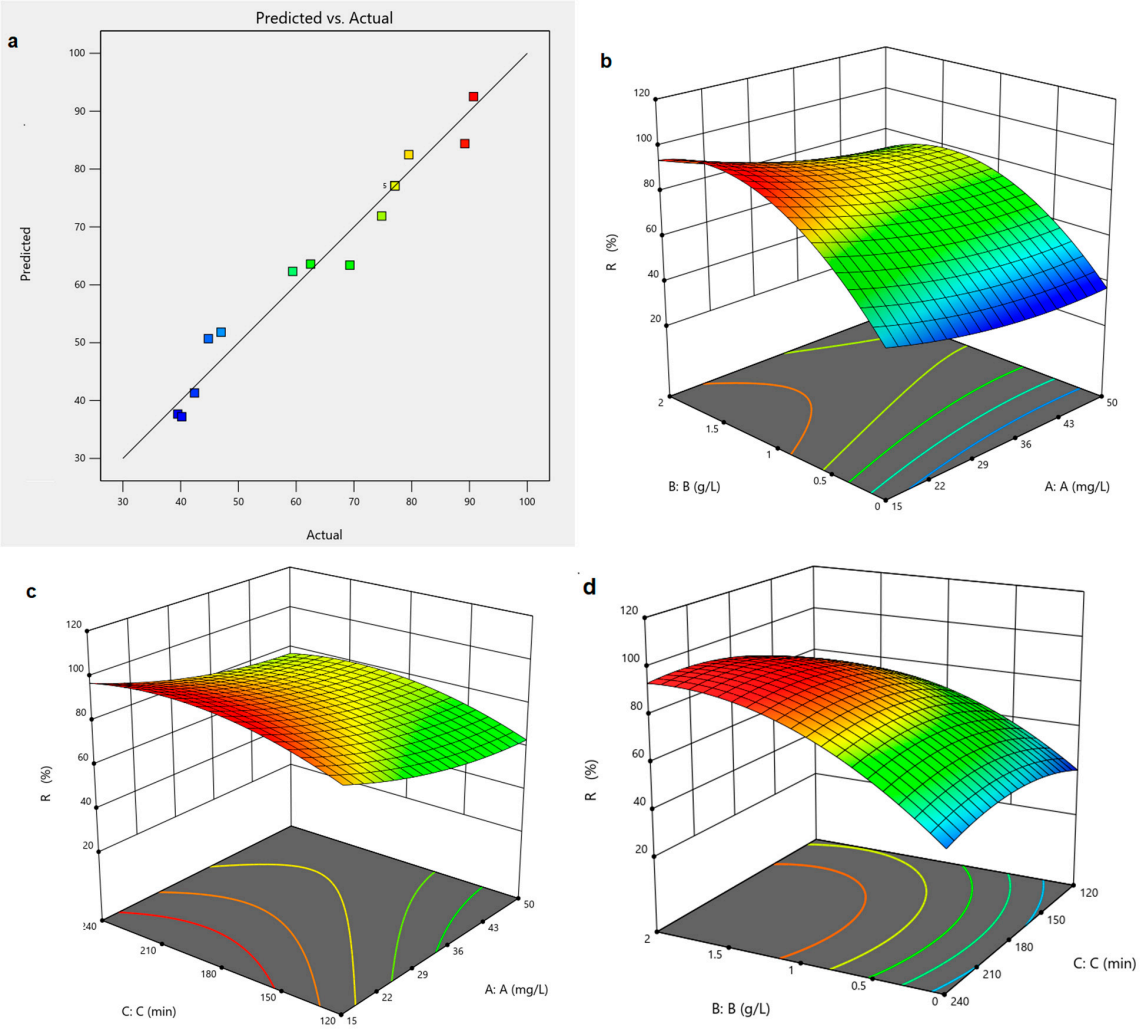
(**a**) Experimental values plotted against the predicted values, derived from the model by using AP2 as photocatalyst. Effects of (**b**) initial concentration of dye and solid–liquid ratio; (**c**) initial concentration of dye and time; (**d**) solid–liquid ratio and time on degradation efficiency of CR dye.

**Figure 9:**
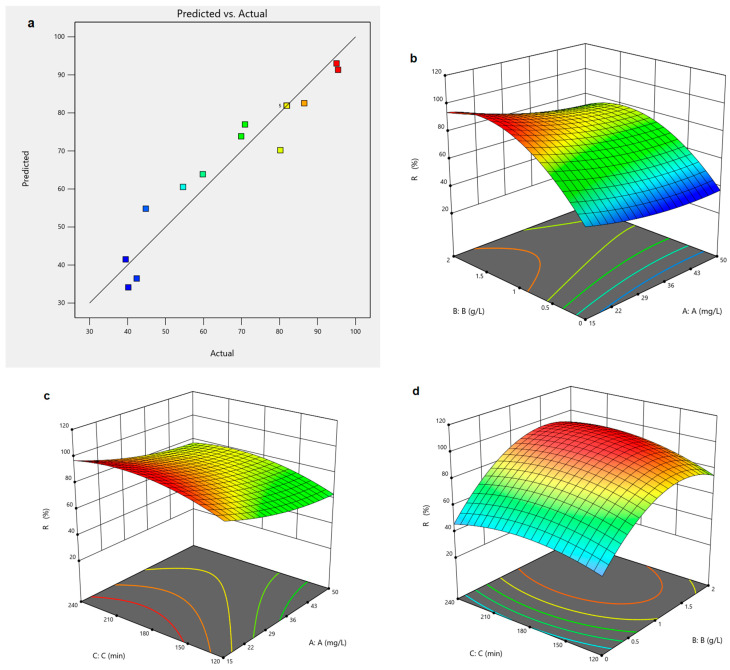
(**a**) Experimental values plotted against the predicted values, derived from the model by using AP2-Zn(II) as photocatalyst. Effects of (**b**) initial concentration of dye and solid–liquid ratio; (**c**) initial concentration of dye and time; (**d**) solid–liquid ratio and time on degradation efficiency of CR dye.

The goodness of the model’s fit is also reflected in the graphical representation of the values of the process efficiencies predicted vs. actual or experimental results, as can be seen from Figure 8a and Figure 9a. The effect of the three independent parameters on the efficiency of the photocatalysis process is also presented by the response surface contour plots in Figure 8b–d and Figure 9b–d. It is observed that, in both cases, in order to obtain maximum efficiency of the photocatalysis process, it is necessary that the initial concentration of the dye be low, the solid–liquid ratio be high, and the reaction time be long.

### 3.6. Photocatalytic Activity

**Figure 10:**
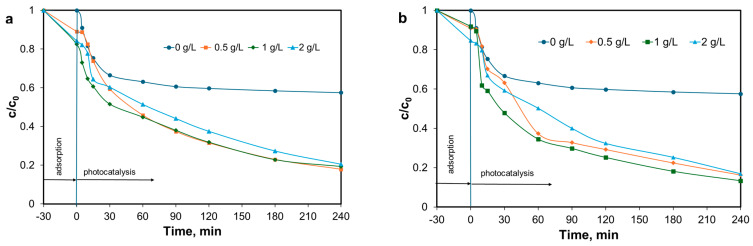
Influence of the S–L ratio on CR removal. (**a**) AP2 photocatalyst; (**b**) AP2-Zn(II) photocatalyst. *c*_0_ = 32 mg/L.

The photocatalytic degradation process of the CR dye was studied by following the variation of the CR concentration over time, depending on (i) the type of photocatalyst (AP2 or AP2-Zn(II)); the solid–liquid ratio (S–L = 0.5, 1 and 2 g/L); the initial concentration of CR (*c*_0_ = 15, 32 and 50 mg/L). Further experiments were performed to observe the behavior of dye solutions upon exposure to light radiation without an added photocatalyst (photolysis). Starting from the initial concentration of CR of 32 mg/L, the decrease in concentration as a function of time was studied for three doses of catalysts (S–L ratio of 0.5, 1 and 2 g/L) compared to the photolysis process, and the results are presented in Figure 10. It is observed that, without the input of catalysts, the process of CR removal (photolysis) proceeds at a high ratio in the first 30 min, and then the concentration of the dye remains almost constant; after 240 min of photolysis, only 42.4% of the initial dye is removed. During the 30 min of dark adsorption, both catalysts retain less than 20% of the concentration of CR present in the solution. Given that the dye oxidation process takes place on the surface of the catalyst, a high degree of adsorption would prevent light radiation from accessing this surface, and the initiation of the dye oxidation process would be prevented. Both types of material used as a photocatalyst, AP2 or AP2-Zn(II), as well as the different doses of photocatalyst, have an influence on the ratio of the CR degradation process, especially in the first part of the process, when the rate of degradation is high. On the other hand, after 240 min of photocatalysis, the degrees of CR removal from water have values of 80% and above, but they are not influenced to a large extent, either by the type of photocatalyst or by its dose.

The times required for the removal of the 50% from the initial concentration of CR by the photocatalysis process (the half-life—t_1/2_) are 60 min, 30 min, and 60 min for the S–L ratio of AP2 catalyst of 0.5 g/L, 1 g/L, and 2 g/L, respectively. When using AP2-Zn(II) as a photocatalyst, t_1/2_ are 45 min, 30 min, and 60 min for the S–L ratios of 0.5 g/L, 1 g/L, and 2 g/L, respectively. Therefore, using an S–L ratio of 1 g/L results in the fastest decrease in the concentration of CR, both when using AP2 or AP2-Zn(II) as photocatalysts.

After 240 min of photocatalysis, the photocatalytic efficiencies of the two materials become similar, and the different S–L ratios no longer play such an important role. Probably, during the photocatalysis process, once the process of generating oxidizing species and initiating radical degradation reactions have occurred, the recombination of electron and hole can increase, resulting in the overall dye degradation process being slowed down. It is observed that slightly lower final yields are obtained if the S–L ratio is 2 g/L, probably because a large amount of catalyst can play the role of a trap for the recombination of oxidizing species.

**Figure 11:**
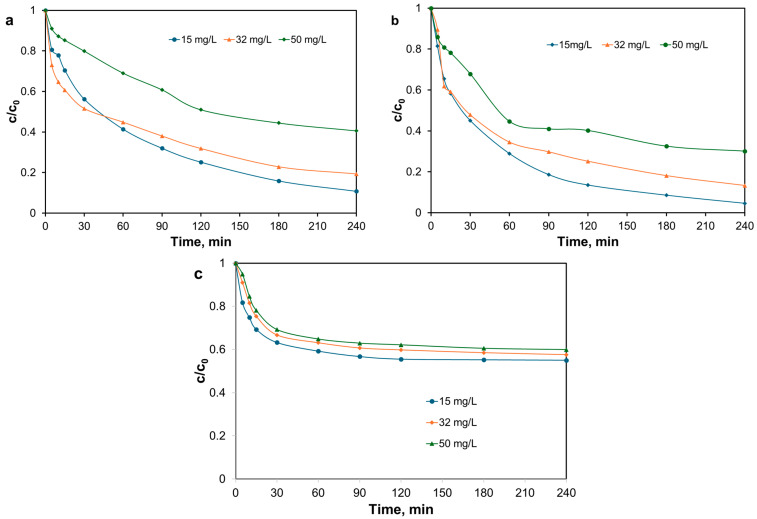
Influence of the initial concentration on CR removal. (**a**) AP2 photocatalyst; (**b**) AP2-Zn(II) photocatalyst. S–L = 1 g/L; (**c**) photolysis.

**Figure 12:**
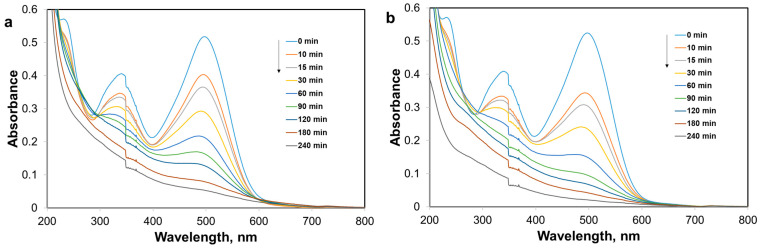
Recorded UV–Vis spectra of the CR solutions during photocatalysis by using AP2 catalyst (**a**) and AP2-Zn(II) catalyst (**b**). *c*_0_ = 15 mg/L, S–L = 1 g/L.

The variation over time of the decrease in initial concentration of the dye was studied for three initial concentrations (15 mg/L, 32 mg/L, and 50 mg/L), and the experimental results are presented in Figure 11 in addition to the results obtained for photolysis experiments. Photolysis experiments show that without a catalyst, the removal of the CR dye from the water has low efficiency, and, after 240 min only, about 40% of the initial concentration has been removed. At low initial concentrations of CR dye of 15 mg/L, after 240 min of photocatalysis, process efficiencies of 89.2% are achieved in the case of the AP2 material and of 95.4% in the case of the AP2-Zn(II) material as photocatalysts. At higher initial concentrations, of 50 mg/L, efficiencies of 59.4% using the AP2 material as photocatalysts and 69.9% using the AP2-Zn(II) material were obtained. A comparison between the behavior of the two photocatalysts in the process of removing CR from aqueous solutions is presented in Figure 12, where the UV–Vis spectra of the solutions recorded during the photocatalysis experiments are presented. It can be seen that, starting from the same initial concentration of CR (*c*_0_ = 15 mg/L) and from the same ratio S–L = 1 g/L, and using the AP2-Zn(II) material, a better efficiency of the process is obtained from the first minutes of photocatalysis. The better efficiency of the AP2-Zn(II) material may be due to the presence of zinc in the structure. Higher initial CR dye concentrations lead to lower rates of degradation as the dye preventing the penetration of light radiation to the surface of the catalysts, and thus the generation of oxidizing radical species, is prevented. However, the photocatalysis process is an advanced oxidation process and could be applied to aqueous streams with low concentrations of dyes so the studied materials can be successfully applied in the tertiary stage of textile wastewater treatment.

In general, the kinetics of the photocatalysis process is described by the Langmuir–Hinshelwood equation:

(5) $$r=-\frac{\mathrm{dc}}{\mathrm{dt}}=\frac{k_{r}K_{c}}{1+K_{c}}$$

where *r* represents the rate of reaction, *c* is the CR dye concentration, and *k_r_* and *K_c_* are constants.

By assuming the condition *K_c_* ≪ 1, and integrating Equation (5) between the limits *c* = *c*_0_ at *t* = 0 and *c* = *c_t_* at *t* = *t*, the Langmuir–Hinshelwood equation is described by the pseudo-first order kinetic model [26]:

(6) $$-ln\left(\frac{c}{c_{0}}\right)=k_{a}t$$

where *k_a_* is the apparent rate constant under experimental conditions (min^−1^).

**Figure 13:**
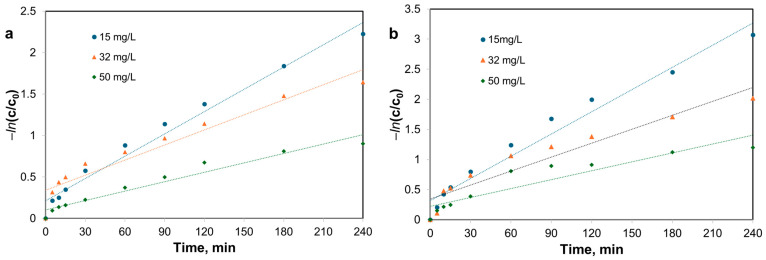
(**a**) Pseudo-first order kinetic of photocatalysis by using AP2 material and (**b**) AP2-Zn(II) material.

The graphic representation of the function $-\ln\left(\frac{c}{c_{0}}\right)=f\left(t\right)$ is presented in Figure 13 (photolysis is excluded for the lack of fit), and the rate constants are presented in Table 2.

From Table 2, it can be seen that photolysis has the lowest rate constants, the process taking place at a higher rate in the first 60 min (see Figure 11c), and then the reaction rate becomes very low. It is also observed that photocatalysis is well represented by pseudo-first order kinetics model (R^2^ > 0.95). The apparent rate constants *k_a_* are higher in the case of using the AP2-Zn(II) material than in the case of using the AP2 material as photocatalysts. Also, the rate constants decrease with the increase in the initial concentration of CR dye.

**Scheme 2:**
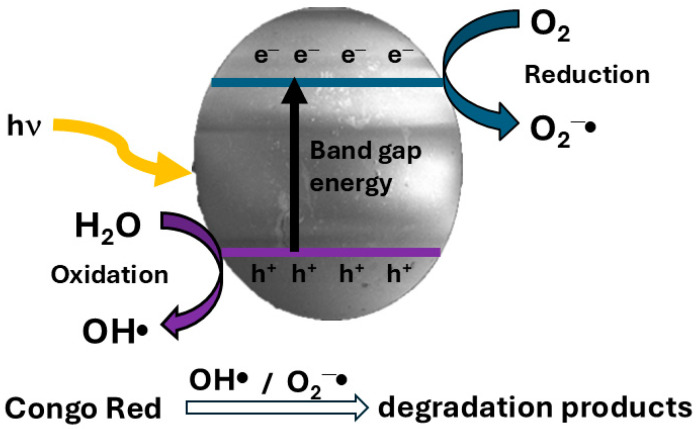
Mechanism for catalytic degradation of Congo red dye.

The mechanism of photocatalysis can occur as follows (Scheme 2): as a result of UV light absorption by the catalyst, electrons (e^−^) are excited to a higher energy level, leaving behind positively charged holes (h^+^). The electrons (e^−^) react with dissolved oxygen (O_2_) to form superoxide radicals (O_2_^−●^), while the holes (h^+^) react with water (H_2_O) to form hydroxyl radicals (HO^●^). The generated reactive species (HO^●^ and O_2_^−●^) decompose the Congo red dye, resulting in degradation products.

A comparison between the experimental conditions and the results obtained from the application of different materials for the removal of CR dye from water is shown in Table 3.

It can be seen that the photocatalysts synthesized and applied by us in the removal of CR dye have similar or even superior efficiencies to other photocatalysts, making them potentially viable for degrading other organic compounds from water.

## 4. Conclusions

A compound of glycine pendant group grafted on styrene-6.7% divinylbenzene (AP2) was produced using a polymer-analogous reaction between chloromethylated styrene-6.7% divinylbenzene copolymer and glycine. By impregnating the AP2 sample with zinc nitrate tetrahydrate, the new compound of type AP2-Zn(II) was also obtained. FTIR and UV–Vis spectroscopy, EDX analysis, SEM, and thermal and textural studies were used to characterize them. To investigate the degradation of CR organic dye, functionalized polymers’ photocatalytic activity was employed. Photodegradation techniques have been applied both to AP2 and AP2-Zn(II) samples. The results of the RSM method show that the dose of photocatalyst and time have a synergistic effect, while the initial concentration of CR dye has an antagonistic effect on the efficiency of the photocatalysis process. The photocatalysis efficiency of AP2 was 89.2%, while the AP2-Zn(II) sample showed a photocatalysis efficiency of 95.4%, after 240 min of irradiation, a catalyst concentration of 1 g/L, and an initial CR concentration of 15 mg/L. The results shown here suggest that the two samples (AP2 and AP2-Zn(II)) might be effectively employed in the process of heterogeneous photocatalysis to remove CR from water.

## Figures and Tables

**Table 1:** Independent parameters and their level.

Variable	Symbol	Units	Level		
			Low −1	Middle 0	High 1
CR initial concentration	A	mg/L	15	32.5	50
Solid–liquid ratio	B	g/L	0	1	2
Time	C	min	120	180	240

**Table 2:** Kinetics parameters obtained for CR photodegradation.

Photocatalyst	*c*_0_ (mg/L)	*k_a_* (min^−1^)	R^2^
Photolysis	15	1.84 × 10^−3^	0.5628
	32	1.90 × 10^−3^	0.6233
	50	1.85 × 10^−3^	0.6300
AP2	15	8.97 × 10^−3^	0.9766
	32	6.06 × 10^−3^	0.9514
	50	3.78 × 10^−3^	0.9537
AP2-Zn(II)	15	12.3 × 10^−3^	0.9656
	32	7.74 × 10^−3^	0.9589
	50	4.93 × 10^−3^	0.9561

**Table 3:** Photodegradation efficiencies of different materials applied in CR dye removal.

Material	*c*_0_ (mg/L)	S–L (g/L)	Time (min)	R (%)	Reference
Y_2_O_3_	27	0.125	360	45	[27]
ZnONPs	20	1	30	99.5	[28]
ZnO-PVP-St	20	0.5	480	72	[29]
Al-SrTiO_3_	10	-	90	81	[30]
AP2	15	1	240	89.2	Present work
AP2-Zn(II)	15	1	240	95.4	Present work

## Data Availability

The original contributions presented in this study are included in the article/Supplementary Materials. Further inquiries can be directed to the corresponding author.

## Supplementary Materials

The following supporting information can be downloaded at https://www.mdpi.com/article/10.3390/polym17050641/s1. Table S1: Experimental design and predicted values for CR removal efficiencies; Table S2: Analysis of variance ANOVA for the response surface model.
