# Supplementary material for: Glycine-Group-Functionalized Polymeric Materials Impregnated with Zn(II) Used in the Photocatalytic Degradation of Congo Red Dye
Source: Polymers (Basel). 2025 Feb 27;17(5):641. doi: 10.3390/polym17050641 (PMC11902704; doi:10.3390/polym17050641)
Supplement: Supplementary file 1 [file polymers-17-00641-s001.zip › polymers-3491536-supplementary.pdf]

Glycine groups-functionalized polymeric materials and impregnated with Zn(II) used in the photocatalytic degradation of Congo Red dye

Laura Cocheci[1*], **Aurelia Visa**[2], **Bianca Maranescu**[3], **Lavinia Lupa**[1], **Aniela Pop**[1], **Ecaterina Stela Dragan**[4] and **Adriana Popa**[2*]

[1] Politehnica University Timisoara, Faculty of Industrial Chemistry and Environmental Engineering, 6 Vasile Parvan Blvd., 300223, Timisoara, Romania; lavinia.lupa@upt.ro; aniela.pop@upt.ro

[2] "Coriolan Drăgulescu" Institute of Chemistry, 24 Mihai Viteazu Blvd., 300223, Timisoara, Romania; apascariu@yahoo.com

[3] West University Timisoara, Faculty of Chemistry, Biology, Geography, Department of Chemistry, 16 Pestalozzi Street, 300115 Timisoara, Romania; biancamaranescu@yahoo.com

[4] Petru Poni Institute of Macromolecular Chemistry, 41A Aleea Grigore Ghica Voda, 700487, Iasi, Romania; stela_dragan@yahoo.com

* Correspondence: laura.cocheci@upt.ro; apopa_ro@yahoo.com; apopa@acad-icht.tm.edu.ro

Table S1. Experimental design and predicted values for CR removal efficiencies.

Run order	Independent variables			Efficiency of CR photodegradation (%) Experimental values		Efficiency of CR photodegradation (%) Predicted values	
	A	B	C	**AP2**	**AP2-Zn**	**AP2**	**AP2-Zn**
1	50	0	180	39.50	39.50	37.68	41.49
2	32.5	1	180	77.10	81.90	77.10	81.90
3	32.5	1	180	77.10	81.90	77.10	81.90
4	32.5	0	120	40.20	40.20	37.21	34.14
5	15	1	120	74.80	86.50	71.89	82.55
6	15	1	240	89.20	95.40	84.39	91.32
7	32.5	1	180	77.10	81.90	77.10	81.90
8	32.5	2	240	69.30	70.90	63.40	76.96
9	50	2	180	79.50	80.20	82.49	70.19
10	32.5	1	180	77.10	81.90	77.10	81.90
11	50	1	120	47.00	59.80	51.81	63.88
12	50	1	240	59.40	69.90	62.31	73.85
13	32.5	1	180	77.10	81.90	77.10	81.90
14	32.5	0	240	42.40	42.40	41.31	36.46
15	15	2	180	90.70	95.00	92.53	93.01
16	32.5	2	120	62.50	54.60	63.59	60.54
17	15	0	180	44.80	44.80	50.70	54.81

Table S2. Analysis of variance ANOVA for the response surface model.

Source	SS		df		MS		F-value		p-value	
	AP2	AP2-Zn(II)	AP2	AP2-Zn(II)	AP2	AP2-Zn(II)	AP2	AP2-Zn(II)	AP2	AP2-Zn(II)
Model	4665.82	5494.68	9	9	518.42	610.52	22.71	10.25	0.0002	0.0002
A	888.31	653.41	1	1	888.31	653.41	38.92	10.97	0.0004	0.0012
B	2281.50	2237.81	1	1	2281.50	2237.81	99.95	37.58	< 0.0001	0.0005
C	264.50	175.78	1	1	264.50	175.78	11.59	2.95	0.0114	0.1295
AB	64.80	22.56	1	1	64.80	22.56	2.84	0.3789	0.1359	0.5577
AC	1.0000	0.3600	1	1	1.0000	0.3600	0.0438	0.0060	0.8402	0.9402
BC	54.76	49.70	1	1	54.76	49.70	2.40	0.8347	0.1653	0.3913
A^2	22.03	82.44	1	1	22.03	82.44	0.9652	1.38	0.3586	0.2778
B^2	794.61	1937.27	1	1	794.61	1937.27	34.81	32.53	0.0006	0.0007
C^2	219.03	298.87	1	1	219.03	298.87	9.60	5.02	0.0174	0.0601
Residual	159.78	416.83	7	7	22.83	59.55				
Lack of Fit	159.78	416.83	3	3	53.26	138.94				
Total	4825.60	5911.51								
R^2 (AP2)	R^2: 0.9669; Adjusted R^2: 0.9243; Adequate precision: 15.0950									
R^2 (AP2-Zn(II))	R^2: 0.9295; Adjusted R^2: 0.8388; Adequate precision: 9.9477									
